# Complete genome sequence of *Bacillus thuringiensis* A1100, producing the selective cytocidal protein Parasporin-5Aa1

**DOI:** 10.1128/mra.00259-26

**Published:** 2026-06-15

**Authors:** Keisuke Ekino, Yuta Nasu, Yuichi Abe, Eiichi Mizuki, Sakae Kitada, Satoshi Harashima, Hiroyuki Saitoh

**Affiliations:** 1 Department of Biotechnology and Life Sciences, Faculty of Biotechnology and Life Sciences, Sojo University 67181, Kumamoto, Japan; 2 Fukuoka Industrial Technology Center, Biotechnology and Food Research Institute, Kurume, Fukuoka, Japan; 3 Department of Bioscience and Bioinformatics, Faculty of Computer Science and Systems Engineering, Kyushu Institute of Technology 221485 , Iizuka, Fukuoka, Japan; Fluxus Inc., Sunnyvale, California, USA

**Keywords:** *Bacillus thuringiensis*, Parasporin, cytocidal proteins, Cry

## Abstract

*Bacillus thuringiensis* strain A1100, producing the selective cytocidal protein Parasporin-5Aa1, carries a 6,123,118-bp chromosome and a 137,009-bp plasmid. The genome was sequenced using Oxford Nanopore and Illumina platforms and assembled using a hybrid approach. This sequence provides a genetic basis for studying parasporin production and its selective cytocidal activity.

## ANNOUNCEMENT

Parasporins (PSs) were discovered among *Bacillus thuringiensis* (Bt) crystal proteins in 2000 and are characterized by selective cytotoxicity against cancer cells (1, 2). Bt strain A1100, isolated from soil at Ryoanji Temple in Kyoto, Japan, produces Parasporin-5Aa1 (PS5). Soil samples were suspended in sterile water, heat-treated at 65°C for 30 min, and plated on nutrient agar at 27°C for 4 days to isolate spore-forming bacteria. Bt strains were identified based on parasporal crystal formation during sporulation observed by phase-contrast microscopy (3). Isolates were maintained on slant cultures in standard agar medium in 3-mL screw-cap vials at 4°C. The isolates were subsequently tested for cytocidal activity against HeLa cells, as described previously (4), and strain A1100 was selected. To better understand the genetic basis of PS5 production and its genomic context, we determined the complete genome sequence of this strain.

A1100 cells were cultured in LB medium at 30°C, and genomic DNA was extracted using standard procedures, including RNase treatment, phenol-chloroform extraction, and ethanol precipitation, followed by quantification with a Qubit 4 Fluorometer (Thermo Fisher Scientific).

For Oxford Nanopore sequencing, libraries were prepared using the SQK-RBK004 Rapid Barcoding Kit (Oxford Nanopore Technologies), loaded onto a FLO-MIN106 flow cell, and sequenced on a MinION device. Base calling was performed using Guppy v6.2.1, generating 148,271 reads (N50, 14,253 bp; total, 1.16 Gb). Reads shorter than 5,000 bp were removed using NanoFilt v2.2.0 (5), and adapter sequences and barcode contamination were trimmed using Porechop v0.2.4 to yield a high-quality data set.

Illumina short-read sequencing was outsourced to Eurofins Genomics (Tokyo, Japan). Libraries were prepared using standard Illumina protocols, and sequencing was performed on the NovaSeq 6000 platform (S4 Reagent Kit), generating 150-bp paired-end reads. This run yielded 1,460 Mb across 9,734,400 reads. After filtering with fastp v0.22.0, 9,381,036 reads (1.40 Gb) were retained with 94.6% Q30 bases. The final genome coverage was approximately 160× for Nanopore reads and 220× for Illumina reads.

A hybrid assembly was generated using Trycycler v0.4.1 (6), incorporating Flye v2.9.1, Raven v1.5.1, and Miniasm v0.3/Minipolish v0.1.2 (7–9), and polished using Medaka v1.4.3 and Polypolish v0.4.3 (10). Genome circularity was determined using the Trycycler reconcile step based on read overlap and consensus assembly. The complete genome consisted of a chromosome and a plasmid. The chromosome was reoriented to start upstream of dnaA as part of the Trycycler reconciliation step, whereas the plasmid was not reoriented due to the absence of a repA homolog. The resulting orientation was independently confirmed using Dnaapler v1.3.0 (11). Genome completeness was assessed using BUSCO v6.0.0 (bacillus_odb12) (12), showing 99.4% completeness and indicating a high-quality genome assembly. All software was run with default parameters unless otherwise specified. The genome features are summarized in Table 1.

**TABLE 1:** Genome features of *Bacillus thuringiensis* A1100

Feature	Value
Genome size (bp)	6,260,127
Chromosome size (bp)	6,123,118
Plasmid size (bp)	137,009
GC content (%)	35.4
CDSs^*a*^	6,423
Ribosomal RNAs	36
Transfer RNAs	106
CRISPR loci	2

^*a*^ CDS, coding sequences.

Genome annotation was performed using the DDBJ Fast Annotation and Submission Tool (DFAST). Notably, the *ps5* gene was located on the chromosome, although Bt toxin genes are typically plasmid-encoded (13). This genome provides a basis for studying parasporin production and Bt toxin evolution.

## Data Availability

This whole-genome sequencing project has been deposited in DDBJ/ENA/GenBank under BioProject accession number PRJDB18305 and BioSample accession number SAMD00794598. The raw sequencing data are available under SRA accession number DRR576721. The complete genome sequences of *Bacillus thuringiensis* A1100 have been deposited in DDBJ/ENA/GenBank under accession numbers AP043659 (chromosome) and AP043660 (plasmid pA1100).
